# Digital ethnography: ethics through the case of QAnon

**DOI:** 10.3389/fsoc.2023.1119531

**Published:** 2023-05-18

**Authors:** Michelle Cera

**Affiliations:** Department of Sociology, New York University, New York, NY, United States

**Keywords:** digital ethnography, ethics, QAnon, privacy, lurking, data fabrication

## Abstract

**Introduction:**

Digital ethnography is a relatively new practice with unclear standards and guidelines. As a result, the ethics of the practice remain unclear. Scholarly debates have emerged surrounding the decision of many researchers and institutional review boards to treat social media data as public. Concerns have also been raised about how informed consent can be adapted to online fieldwork. How does a researcher make their presence known when they are not visible in the traditional sense? Which online interactions should be considered public, and which are private? How can we protect the anonymity of social media users?

**Methods:**

This article leverages original digital ethnographic research on QAnon social media spaces to suggest ethical guidelines for digital ethnographic practices.

**Discussion:**

It begins with a description of the research, followed by discussions of the public-private binary, lurking, data reconstruction, and institutional review boards.

**Results:**

This article advocates for rethinking the public-private binary as it applies to the digital world, ameliorating the “lurker” concern by making the presence of the researcher known in appropriate spaces, and maintaining the integrity of the data by avoiding reconstruction. Although many digital ethnographers have chosen to reconstruct or paraphrase online data to protect privacy, this practice comes with its own ethical dilemmas. The ethical dilemmas and guidance discussed in this article are critical lessons for digital and in-person ethnographers alike.

## 1. Introduction

Digital ethnography, sometimes referred to as virtual ethnography, ethnography of cyberspace, and online ethnography, is a relatively new set of methods within the social sciences. It typically adapts offline methods such as participant observation and interviews to online space. This new terrain poses important methodological and ethical challenges. For example, how and from whom should informed consent be obtained in social media groups with thousands of users? When can a researcher reasonably assume a post is public? How should we think about privacy, anonymity, and the prevention of harm online?

This article focuses on three debates present in existing scholarship on digital ethnography: the public-private binary, data fabrication, and the “lurker” concern. Diverse perspectives and varying interpretations characterize each topic. Researchers continue to struggle with when to classify data collected online as public or private. Their decisions have implications for the wellbeing of participants. Many scholars have suggested data fabrication, or in other words paraphrasing easily searchable data collected online, to protect privacy and anonymity. Relatedly, because digital ethnographers are not visible in the traditional sense, they need to be careful to avoid “lurking” in digital spaces. They must decide when and how to make their presence known and participate in interactions. Additionally, this article considers the role of institutional review boards in evaluating digital ethnographies. I use the case of QAnon to suggest these dilemmas can largely be ameliorated through transparency, attention to context, the use of real data, and open discussion of the research process rather than covert methods.

After a discussion of QAnon and the specifics of my digital ethnography, I divide the article into four sections. First, I think through the public-private debate and argue this binary should be considered a spectrum based on barriers to accessing digital spaces. The next section addresses lurking and how to ensure active participation and transparency. I then show how data reconstruction (or “fabrication”) poses its own ethical challenges and suggest alternatives. Finally, I explore my own experiences with institutional review boards and offer suggestions for digital ethnography proposals. All four sections leverage my research on QAnon to address the complexities of each debate.

Although this article reflects on ethical issues relevant to digital ethnography, it addresses age-old debates about in-person ethnographies as well. Duneier et al. (2014) emphasize enduring questions about urban ethnography that can to be adapted to online space: *How* did the researcher develop their relationship with their subjects? What is the nature of their relationships, and how should they be managed? What is the standpoint of the observer? How can we be more open about the research process? My suggestions for ethical practice should be thought of in relation to the enterprise of ethnography itself. I draw on two perennial issues in ethnography: the ethnographic relationship and the influence of the local. Traditional ethnography is defined by the relationship between the researcher and the researched. It has also always been rooted in local contexts. Accordingly, digital ethnographers should incorporate and reflect on the importance of their relationships and the particularities of their case and contexts as part of ethical practice.

Further, QAnon is a useful case through which to think through general ethical issues with digital ethnography. The extreme nature of the group makes it particularly necessary to develop and uphold strict ethical standards. For example, QAnons often use violent or otherwise disturbing rhetoric. This forces the researcher to grapple with when and how to reproduce data in publication. It also means researchers need to be more acutely aware of their own safety within digital spaces. QAnons are also largely anonymous, complicating the kind of relationships which can develop with the researcher as well as increasing expectations of privacy. The nature of the group opens up pressing ethical questions which might not have surfaced in an investigation of more moderate groups.

## 2. A digital ethnography of QAnon

QAnon emerged on October 28th, 2017, when an anonymous account called “Q” posted a cryptic message about an oncoming “storm” on the social media site 4chan. Q and QAnons have since been responsible for the production and spread of conspiracy theories throughout social media, including the idea that a satanic cabal of lizard-like politicians control politics, media, and other institutions. It has also been linked to offline events such as January 6th and the “Pizzagate” shooting in Washington D.C. Among the American electorate, roughly 17% agree with the QAnon conspiracy theory that a satanic group of elites run a sex trafficking ring and control politics and media (Staff, 2021), 7% have a favorable view of QAnon overall (Schaffner, 2020), and 8% of registered Republican voters identify as a QAnon “supporter” (Civiqs, 2020).

I began a digital ethnography of QAnon in January of 2022. My research design was inspired by scholars developing the craft (see, for example, Boyd, 2014; Markham, 2017; Lane, 2019; Stuart, 2020). Boyd (2016) suggests digital ethnography is much like traditional ethnography, in that it should include deep immersion in your field site(s), participant observation, content analysis, and semi-structured interviews. Although the research was conducted in a largely inductive manner, I approached my field sites with several questions: What repertoires, logics, and practices makeup QAnon political participation? How should we classify QAnon as a group, and therefore understand their activity as a whole? How do QAnons move through social media space? My goal was to understand the historical and technological specificity of QAnons' participation in politics.

Field site selection was the first difficult task. Boyd (2008) and Beneito-Montagut et al. (2017) both argue that digital ethnography requires rethinking what counts as a field site by moving beyond a bounded sense of place. Social groups exist within and between multiple platforms and groups simultaneously. I began the process by directly messaging users on various platforms I knew were home to QAnons and other far-right groups, such as MeWe, 4chan, and Telegram. I asked them which platforms they prefer to use, as well as where they see most QAnon activity occurring. I then Zoom-interviewed a QAnon member with an outsized presence online.^1^ “Operation Q,” as he calls himself, suggested MeWe, Telegram, the chans (4chan and 8kun), and Telegram. I continued to ask about platform preference in video-chats and direct messages with other QAnons, finally settling on five major platforms: Gab, Telegram, 4chan, 8kun, and MeWe. I selected three groups within each platform based on their size (excluding groups with fewer than 10,000 members), frequency of activity, and of course affiliation with QAnon.

Deep immersion in these digital communities involved behaving “in the same manner as my informants” (Bluteau, 2019, p. 268). I learned the norms and unique subcultures of each space to the best of my ability. Although many researchers choose to hide their identity when conducting research on extreme groups, I decided to use my real name, photos, participate with my real perspectives, and disclose my position as a researcher. I let participants know I was conducting research on their political participation. I felt this was an important ethical practice. After the first few weeks, I understood the protocol of each space well enough to participate more regularly. I began to comment on posts with my own thoughts and questions. I posted links to articles on relevant current events.

Content analysis involved pulling the “top” posts (most likes, comments, etc.) from each group every time I logged on. The goal of content analysis was to see what kinds of topics are discussed, how they are discussed, who discusses what, what content is particularly popular, and the emotional tone of the posts.

Interviews^2^ were conducted over Zoom, FaceTime, email, and direct messages. Oftentimes, interviews would include screensharing with willing participants who would walk me through their daily social media activity, an approach suggested by Ardévol and Gómez-Cruz (2012) as well as Light et al. (2018). Participants who were particularly concerned about their anonymity decided to message back and forth with me rather than disclose their identity through phone calls or video chats. These “interviews” were essentially ongoing, as we continued to go back and forth for weeks even after we had signed off for the day. Interviews were conducted primarily to understand meaning-making behind interactions I had observed as well as how participants viewed their own activities and purposes.

## 3. Public vs. private

Matzner and Ochs (2017) remind us that debates about what counts as private and public go as far back as Ancient Greece. Ethnographers have long grappled with participation in public and private spaces, especially given deviance, social disorganization, and suffering are often the focus of ethnographic research (Katz, 1997). Traditional ethnographers have had to balance making their presence known as a matter of ethics while trying to get “behind the scenes” perspectives. Howard Becker has written extensively on sociologists balancing public and private spaces. He believed sociologists of his time still had no consensus as to what data can and should be made available to the public (Becker, 1974).

Today, those interested in digital ethnography typically look to the Association of Internet Researchers (AoIR, 2020). The AoIR has become industry standard. The international organization provides regularly updated guidelines for scholars who conduct research on the internet. The third edition of the guidelines, published in 2019, focuses on three primary issues: ethical pluralism, informed consent, and privacy. Similar to Nissenbaum (2011) and Eynon et al. (2016), the AoIR recommends treating ethics as a case-by-case approach to take into account the values and perspectives of other cultures. Informed consent, and ethical considerations overall, should be considered a process rather than a box to check at the beginning of the research process. Some researchers may choose to apply pseudonyms, some delete identifiable information, some avoid asking sensitive questions, and some only ask for consent at the dissemination stage. All decisions need to be contextual and relational. Privacy is discussed in relation to publicity, and the guidelines suggest the greater the acknowledgment of publicity, the less need there is for anonymity and confidentiality. However, the guidelines do not specify how a researcher determines the acknowledgment of publicity.

Internet researchers now have to contend with perrennial public vs. private debates as they apply to our online social worlds. Digital research often heightens the risks of privacy violations (Marres, 2017). Approaches vary significantly both within and between disciplines. While some argue everything on the internet is public and can therefore be used by researchers without informed consent, others take special care to inform each and every participant about the use of their data.

Kitchin (2003) takes the former approach. She reflects on two important issues relevant to digital ethnography: (1) What constitutes public and private on the internet? and (2) Should internet participants expect confidentiality and anonymity? She suggests that because the internet is by definition a public space, participants cannot expect confidentiality or anonymity. Accordingly, informed consent is unnecessary. Sugiura et al. (2017) ask similar questions. Should researchers use publicly available data on the internet without informed consent? Is informed consent feasible online? How can anonymity be preserved? They review several organizations whose purpose is to provide ethical guidelines. The Marketing Research Association (2010) guide to social media research suggests most participants understand their conversations are public, but most do not expect their data to be viewable to researchers. Yet they maintain informed consent is not needed because of the public nature of the interactions online. The Council of American Survey Research Organizations (CASRO, 2011)'s social media guidelines state informed consent should be obtained when research participants directly interact with researchers. They do not specify what constitutes direct interaction.

Boyd (2008) finds her participants struggle with defining public and private. There are cultural differences in how individuals and groups understand privacy, and individual-centered notions of privacy obscure the contextually-mediated nature of digital spaces as well as the role of technical affordances. Marwick and Boyd (2014) refer to this concept as “networked privacy.” Nissenbaum (2011) also argues privacy is contextually mediated: local expectations and norms dictate expectations of privacy. Although individuals might post on a public site, they might not understand their content is public. Ultimately, we need to be diligent about how *participants* define public and private (Boellstorff et al., 2012). These arguments can and have been applied to other ethical considerations, and many have argued for sensitivity to cultural differences throughout the whole research process (Hongladarom, 2017; Hutchinson et al., 2017; Luka et al., 2017; Weller and Kinder-Kurlanda, 2017).

Yet researchers still need to develop working definitions of public and private to ensure the protection of human subjects online. Boyd (2008, p. 21) ultimately defines public as: “A space where people may gather, interact, and be viewed and also an imagined community of people who share similar practices, identities, and cultural understandings. That which is public is potentially but not necessarily visible.” She highlights that public spaces might not be visible, but then how should researchers approach the more invisible spaces? Further, her definition speaks to a broad range of online spaces, such that groups, newsfeeds, direct messages, and more can be considered public. Some differentiation is necessary to guide ethical practice.

This differentiation need not be binary (e.g., all of x is public, and all of y is private). For example, Reilly and Trevisan (2016) call facebook “semi-public.” Eynon et al. (2008) refer to some spaces as “in between.” There is no clear resolution as to what is public and what is private online. What questions can we ask ourselves, and what guidelines can we follow to prevent harm to participants?

### 3.1. QAnon and the public vs. private debate

It is increasingly difficult to distinguish between public and private on social media as platforms develop new features and ways of communicating. Some QAnon researchers approach this debate by only using data publicly available to anyone with internet access (see Papasavva et al., 2020; Hanley et al., 2022; Kim and Kim, 2022). Others involve direct interaction with QAnons through interviews and surveys, and therefore necessarily use private information (see, for example, Garry et al., 2021). Researchers may even choose to use indivdual “user bios” which include personal information about gender, race, and other identifying factors (see Bär et al., 2022). To further complicate the matter, expectations and understandings of privacy vary not only nationally and culturally, but also by social media subculture. My research on QAnon has made it clear that public and private should not be treated as binaries, but rather as a spectrum.

**Figure 1:**
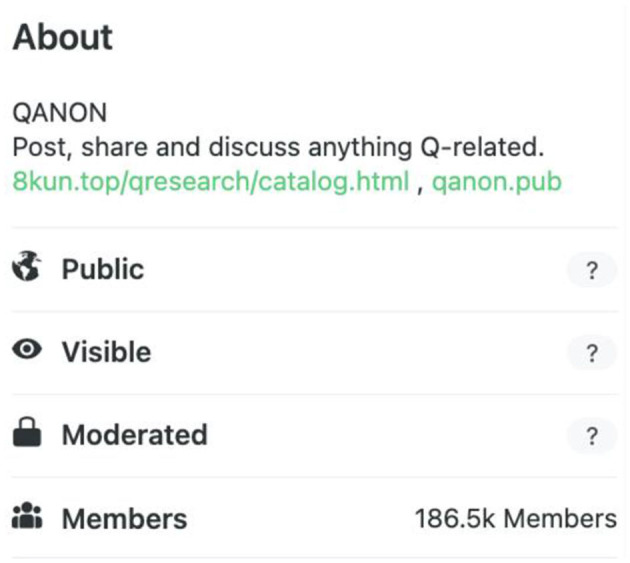
Gab group display.

QAnon presents an interesting case through which to think through issues of privacy. Their group is built around anonymity and secrecy. The supposed head of QAnon, Q, is unknown to their followers. Nearly all accounts following Q-related groups on 4chan, 8kun, Gab, and Parler (four of the most frequented sites by QAnon adherents) are anonymous. For example, three regular posters in the “QAnon” group on Gab are @TruthRevealer17, @TheStormIsReal, and @TheBigVirusHoax. All of their posts and profiles are viewable by all users of Gab. Moreover, each platform signals different levels of privacy. “QAnon” on Gab displays the following at the top of the group (Figure 1). If you mouse over the question mark next to “Public,” Gab indicates, “Anyone on or off Gab can view group posts.” If you mouse over the question mark next to “Visible,” it tells you “Anyone can find this group in search and other places on Gab.” Private groups must be requested to join and users are accepted or denied by administrators. They are labeled “Private,” and the question mark indicates, “Only members can see group posts.”

**Figure 2:**
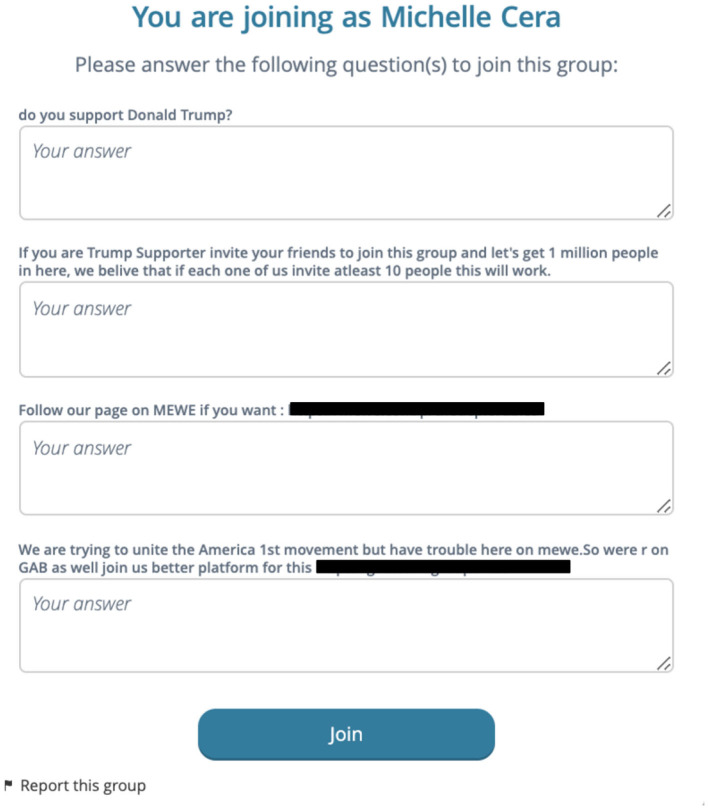
MeWe questionnaire.

MeWe groups signal privacy in a different way. While many groups are public, some require users to fill out a questionnaire which is reviewed by administrators. Figure 2 below is a questionnaire issued by a private, far-right MeWe group known for promoting QAnon conspiracy theories. Questionnaires like these came up with about half of the QAnon groups I have joined on MeWe. They act as barriers to entry and signal to others that their content is not meant for public consumption. Yet once I filled out the questionnaire and told them I do not in fact support Donald Trump, I was still allowed in the group. Is the group then semi-public? Mostly private?

I now have accounts on dozens of QAnon-frequented platforms, including Telegram, Parler, MeWe, 4chan, 8kun, and Gab. Each platform offers different forms of privacy. Moreover, each platform offers different ways of communicating with each other. Some support private messages, some have chat rooms, some have groups, others are based on hashtags, and on. These features vary not only by platform but within platforms, and are constantly adjusted by companies seeking to make the most profitable products for users. Much like offline ethnographers suggest, local context matters. Therefore, it makes little sense to universalize a definition of public and private online.

Instead, we should treat public and private as falling on a spectrum. This directly contradicts advice from Kitchin (2003, 2007) who suggests all content found on the internet is by nature public. Instead, it aligns with the work of communication scholars such as Scott (2013) who argues classification frameworks should be oriented away from binaries. Concepts such as identity, transparency, and collectivity fall on a spectrum. Nearly all social media spaces will fall somewhere in between public and private. The spectrum should vary based on barriers to access (such as questionnaires discussed above). In other words, the more difficult it is to access a social media space, the more private it becomes. If anyone with an internet access and account can access a space, it falls more on the public side of the spectrum. The more difficult a space is to access and therefore the closer it is to being “private,” the higher our ethical standards need to be. I have not pulled any data from the group requiring a questionnaire without directly messaging users and obtaining informed consent. I have never published direct messages with participants without informed consent. Small social media groups with <50 members should always be informed of the presence of a researcher. I feel it unnecessary to obtain informed consent for posts made by celebrities in more public groups or feeds. And for groups with hundreds of thousands of members, I typically feel it unnecessary to inform each member of my position as a researcher. “Walking through” social media apps with participants, as discussed in the methods section, can also be useful in determining how public or private participants consider their activity. Direct discussion with participants about privacy concerns strengthens the relationships that are necessary to produce accurate and meaningful portrayals of their lives, and these relationships and discussions are equally important in determining ethical practice.

The case of QAnon offers insights that can contribute to the resolution of current debates within digital research. The public vs. private debate is ongoing, and some researchers suggest all content on the internet is by definition public. Others suggest thinking of privacy on a case by case basis. Researchers have not developed guidelines for how to determine ecactly what is public online. Some argue informed consent is not needed in internet research because of the public nature of interactions, others emphasize cultural variations in expectations of privacy. Eynon et al. (2008) and Reilly and Trevisan (2016) somewhat hint at a privacy spectrum with phrases such as “semi-public,” but this argument has not been fully fleshed out. My research on QAnon has led me to think of publicity and privacy not as a binary but as a spectrum based on barriers to access. Rather than using a universal definition of public, researchers might think about how difficult a digital space is to access to determine the nature of the space. Platform context is key here, as different platforms offer different barriers and indications of privacy. The following questions are useful: How many members are within the space? How many “private” signals are present? How many barriers to access are present? Is there a way to determine user expectations of privacy? These questions have proven useful in guiding my own practice within QAnon spaces, and will hopefully be of use to other researchers.

## 4. Lurking

Lurking, a practice in which reserachers observe social worlds and extract data without informing participants of their presence or use of their data, has plagued online and offline ethnographers alike. Some offline ethnographers believe covert observation can be beneficial to data collection and interpretation, especially in cases where the communities are difficult to access or vulnerable (Ellis, 1995; Becker, 1998; Humphreys, 2017). On the other hand, Lareau (2003) argues relationship building as well as the development of trust and comfort are crucial to ethnographic practices. Lurking by definition prevents meaningful relationships, trust, and comfort. Some offline ethnographers have approached these issues by involving participants more directly in their research processes. For example, Venkatesh (2002) had his participants interpret his research practices and narratives in informative ways.

Like offline ethnographers, lurking has been a central concern of digital ethnographers for some time. Researchers can enter digital worlds easily and without notice, as many social media platforms afford anonymity. Varis (2014) suggests the data gained by lurking cannot be considered digital ethnography, as the “participant” aspect of participant observation is missing. Part of the principles of informed consent is ensuring people comprehend what ethnography is and what researchers are doing, beyond a simple form (Boellstorff et al., 2012). Scholars have begun to address these issues and how they can be avoided. Kaufmann and Tzanetakis (2020) argue permanent connectivity of spaces on the internet allows the researcher to always be co-present. This first-hand, in-the-moment experience is crucial for qualitative research. Digital spaces also allow you to talk to people you might otherwise not be able to talk to, and they might divulge things they would not in person. Yet they note this type of research becomes complicated when studying vulnerable populations. They avoid lurking by making participants “co-researchers,” or in other words allowing them to make sense of their own data when possible. Marres (2017) make an important argument on this subject: digital platforms make it easier for researchers to involve their participants throughout the entire process. Platforms afford new and plentiful forms of interaction with participants, enabling research processes based heavily on *exchange*.

Eynon et al. (2008) specifically discuss observation of online communities. Strategies they suggest include approaching key stakeholders of groups to ask permission and visibly labeling yourself as a researcher (such as in your profile or username). They also suggest being explicit about ethical dilemmas and decisions in publications. Boellstorff et al. (2012, p. 142) also recommends being honest and upfront about identity, as “undercover observation… runs counter to the heart and soul of ethnography.”

Hine (2000) was one of the first to problematize digital lurking. She later broadened the range of activities which should be considered participation, including browsing, following links, and moving between platforms (Hine, 2007). de Seta (2020, p. 87) introduces the notion of “participatory modalities” to undermine the seriousness of lurking. He argues participation can vary from non-use to active presence, none of which should be considered lurking.

On the other hand, lurking may be beneficial for the quality of the data obtained by the researcher. Grincheva (2017) acknowledges that lurking is a major issue but contends the practice allows you to observe participants in a more natural setting. While a researcher in offline settings has no choice but to affect the interactions they observe, the online researcher can observe interactions as they would take place in their absence. Grincheva still cautions against lurking unless participants understand their activity is accessible to the whole internet and not just their group.

### 4.1. QAnon and the lurking debate

Lurking has been defined differently by various researchers, but the principal concern is that social media study participants are unaware they are being observed and uninformed as to the use of their data. QAnons can be considered a vulnerable population given their status as an extremist group, potential for violent or unpredictable behavior, and the risk of losing employment or social ties should their beliefs be disclosed. Therefore, I find it particularly important to avoid lurking in their digital spaces. While I do not endorse their extreme views, I maintain that ethical standards must be applied universally.

Perhaps most controversially, I use my real name and photo in all of the QAnon spaces I study. Most QAnon researchers choose to hide their identity, yet some choose disclose their position as a researcher and are candid about their beliefs (see Forberg, 2022). My research participants deserve to know my real identity and purposes, regardless of their discriminatory political views or the fact that they are typically anonymous themselves. Transparency helps build relationships which are crucial to ethical and accurate research. It can be exploitative to mislead study participants on your intentions. I grant all participants the same respect I expect to receive in return. I cannot expect participants to be forthcoming on the various personal and political discussions we have if the premise of the research is dishonest. I have often found study participants appreciative of my honesty and sometimes more willing to speak with me candidly in interviews.

**Figure 3:**
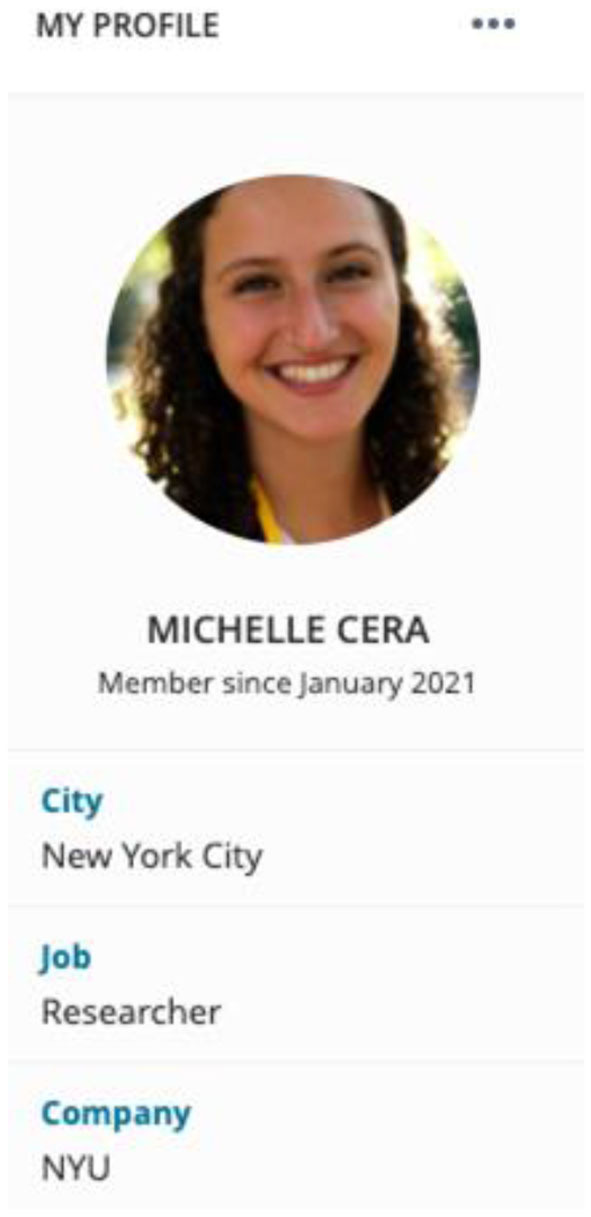
MeWe profile.

Figure 3 is a screenshot of what my profile looks like on MeWe. I use a real photo, my real name, the city I live in, and indicate my position as a researcher and my institutional affiliation. Anyone who navigates to my profile can easily view these credentials because my profile is public. Further, I inform study participants of my research intentions in direct messages, group chats, questionnaires, and interviews. QAnons are particularly active in direct messages as one of their main goals is to spread information. For example, on MeWe, I receive upwards of 50 messages per day in direct messages or in group chats of spaces such as “WWG1WGA.” Whenever a QAnon reaches out to me personally or when I reach out to ask questions or request an interview, my first message indicates my name and my research intentions. I find I am able to get quality data not by hiding my identity but by being forthcoming.

**Figure 4:**
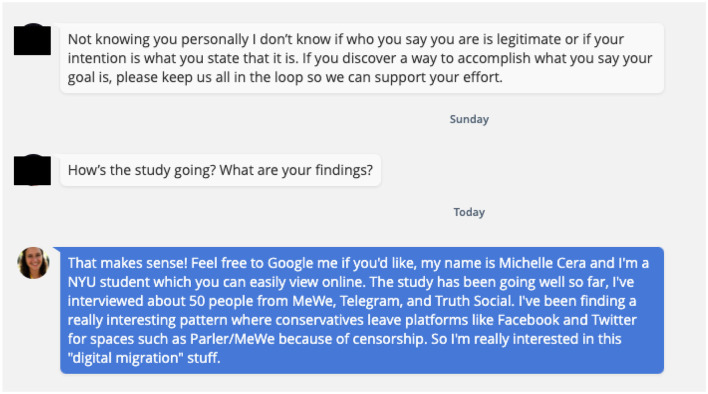
Chat exchange.

The chat exchange below (Figure 4) is typical of my conversations with QAnons. Most users are immediately suspicious of me for living in a liberal city and being affiliated with academia, an institution most of them despise. After receiving a request for an interview in a direct message, Susan (a pseudonym) tells me she has reason to be skeptical of my identity and intentions. However, she indicates support for my research. I respond by suggesting she Google me to confirm my identity, and discuss one of my findings honestly. Susan then agreed to be interviewed at a later date. My interactions with Susan underscore the power of transparency in digital research.

Lurking should be especially avoided within small groups of up to 50 users. In my experience, this tends to be the cut off at which members stop recognizing most other members. In small groups, users tend to feel safe to discuss personal views and information. I have often come across discussions of drug use, marital issues, and health concerns in small groups. Users are also more likely to get to know each other, unlike in groups of tens or even hundreds of thousands of users. Small groups typically have more targeted purposes for select users in comparison to large groups which tend to be curated for public audiences. Therefore, small groups fall farther on the “private” side of the spectrum. Accordingly, researchers should take into account the size of the group when deciding how to approach participants and observe their spaces. I tend to announce my presence in a post viewable to all members of small groups. I do not do this in larger groups as the likelihood all members view my post or care is lower.

Avoiding lurking is also about understanding how to ensure the “participant” part of participant observation. de Seta's (2020) participatory modalities underscore the various ways in which a researcher can be active in the communities they study. While announcing one's presence and intentions is a first step, participation is a continuous practice. Commenting, liking (or disliking) content, posting text, articles, or memes, and messaging users are all forms of participation. Another way to meaningfully participate in the digital social worlds we study is to engage participants more deeply in the research process, from developing questions to data interpretation to publication.

The key is to digital and offline participant observation is to engage in a similar manner as other users within a space. This requires learning the norms and practices of each platform and group. For example, Marwick and Partin (2022) engage in what they call “deep hanging out,” a form of observation where researchers monitor and spend extended periods of time in a group. Forberg (2022) similarly consumes content within groups for long periods of time, but also conducts interviews with participants. Conner and MacMurray (2022) primarily rely on content analysis and video observation.

The lurking debate endures. While many researchers believe lurking might lend itself to higher quality data, others argue lurking goes against the very definition of ethnography. Very few researchers have chosen to use their real identities, especially within extremist groups. I argue lurking is contrary to the methods of ethnography itself. I am candid about my identity because it is an important ethical practice for all researchers, but I have also found it useful in developing trust and relationships with participants. Much like the private and public spectrum, the need for transparency should increase as barriers to entry increase. The more difficult it is to enter a space or the smaller it is, the higher the need to identify yourself as a researcher. When ethnographers lurk, they lose the richness of relationships that are crucial to the endeavor of the method. They misrepresent their purposes and in doing so leave room for the distortion of their findings, relationships, and interpretations. I argue researchers should use their real names and indicate their positions when appropriate, participate in multiple ways, engage in a manner similar to other users in the space, and be forthcoming about the purpose of their studies.

## 5. Data fabrication

Data fabrication is the process by which researchers paraphrase or change the original speech or text produced by the study participant to avoid the participant being traced upon publication. Although digital ethnography has resurfaced these issues, offline ethnographers have always had to contend with the fair and accurate presentation of their data. Decision-making in regards to who should be quoted, when, and how has always affected offline ethnographers. Several notable ethnographers have written extensively about the challenges of protecting participant confidentiality, especially for those in vulnerable communities (Scheper-Hughes, 1993; Nakamura, 2006; Agustín, 2008; Desmond, 2016). While they do not address reproducing quotes themselves, they do address how recreating and representing data leave room for harm. Further, presentation of data is intimately tied to positionality for offline ethnographers (Collins, 1986; Smith, 1990; Borgois, 1996). Ellis (2004) underscores how researcher biases and assumptions shape the narratives they tell in the publication of their data. These questions are uniquely challenging in online contexts.

The use of traceable or searchable data in the social sciences is thought to be one of the biggest concerns for digital researchers (Kling, 1996; Beaulieu and Estalella, 2012; Townsend and Wallace, 2016; Lane and Lingel, 2022). As digital tools become more robust, so too do the risks of our participants being located and searched (Shklovski and Vertesi, 2013). The most recent edition of the AoIR guidelines also addresses this issue. Those who opt for data fabrication argue our primary goal as researchers is to protect the privacy and anonymity of our participants. Boellstorff et al. (2012) argues the most important thing to keep in mind are the consequences of participants being identified.

Screenshots, videos, and audio recordings taken from online spaces are particularly vulnerable to privacy violations (Boellstorff et al., 2012). Accordingly, many social media scholars argue we should paraphrase this kind of data and have done so in their own work. Boyd (2016, p. 91) tells us, “When I quote text from profiles, I often alter the quotes to maintain the meaning but to make the quote itself unsearchable.” Mukherjee (2017) studies a vulnerable community: victims of Intimate Partner Violence (IPV). She paraphrases all data she uses in publication to ensure the safety of her participants. Markham (2012) suggests fabricating posts can be done in such a way that is true to the broad themes of the data. de Seta (2020, p. 91) takes it a step further:

“Fabrication is thus inextricably linked to the idea of expertise. In claiming and embracing one's role as editor, translator, and fabricator of multimedia and multimodal vignettes, of composites of events, identities and inscriptions, the digital ethnographer implicitly establishes competence and knowledgeability over a certain sociotechnical context.”

Yet how can a researcher establish knowledgeability over a social world while also distorting the content it produces? For this reason, some QAnon scholars choose to use direct screenshots without alteration (see de Zeeuw et al., 2020; Hanley et al., 2022).

Other scholars suggest alternative methods to avoid the issues that arise from data fabrication. Sugiura et al. (2017) argue removing identifying data is not enough because search engines utilize advanced technology to trace data, and therefore we should summarize the data instead. Boellstorff et al. (2012) alters details such as the time and place of events, and takes care to avoid particularly loaded scenarios. Shklovski and Vertesi (2013) avoid searchability by “Un-Googling,” a practice where they remove all identifiers, including names, titles, and contextual details of the environment including city and country names. Reilly and Trevisan (2016) only quote directly from public figures because they do not have the expectation of privacy or anonymity. They also use word clouds to show the most commonly used words in posts rather than the posts themselves. And while Robson (2017) sometimes paraphrases the data, he typically puts direct quotations into narrative form.

Data fabrication ultimately speaks to a much broader issue within digital research, the “crisis of representation,” which is our decreased ability to adequately represent society (Marres, 2017). Researchers are less able to “establish knowledgeability” over digital spaces given anonymity and other features of digital interaction which prevent accurate representation. However, data fabrication has become an increasingly common practice within social media research. The implications of this practice are substantial. Inaccurate portrayal of the data has myriad consequences. When a researcher paraphrases or alters the original speech or text, the door is left open to bias and distortion of meaning. The integrity of the data is more compromised the farther the text gets from its original form. Word clouds and narrative summaries do not necessarily solve the problem. Care must be taken to avoid harm to participants, and especially to vulnerable participants such as the communities studied by Mukherjee (2017), yet care must also be taken to stay true to the data produced within the social worlds we study.

### 5.1. QAnon and the data fabrication debate

Data fabrication, or the process by which researchers alter the original text of an online post to avoid traceability, is preferred by many digital media scholars. The Google Age makes it easy to search for and find content and its associated author or authors, including not just text but images with the reverse image search option. Yet when researchers reconstruct digital data produced by study participants, they leave room for the alteration or mischaracterization of meaning.

This issue became immediately apparent when I began to study QAnon. QAnons post purposefully cryptic messages which are meant to be decoded by insiders. They also frequently “troll,” or in other words post insincere and inflammatory messages. They have developed their own language and each platform and group have unique subcultures. Outsiders simply do not have the cultural knowledge to meaningfully reproduce primary data, and even if they do, there is no way to determine the intention of the poster.

There is also no way to determine the identity of a poster. Social media platforms make it easy to present fake profiles, names, photos, and more. QAnons disproportionately remain anonymous online (hence their name). Therefore, researchers have no meaningful way to discern age, race, gender, or other attributes social scientists find important. Analysis then becomes particularly difficult because researchers cannot determine how perspectives might be influenced nor draw out meaningful patterns among social groups. In line with the “crisis of representation” (Marres, 2017), the issue of unknown identities places the researcher at a farther distance from their study participants, making it difficult to faithfully represent their data.

**Figure 5:**
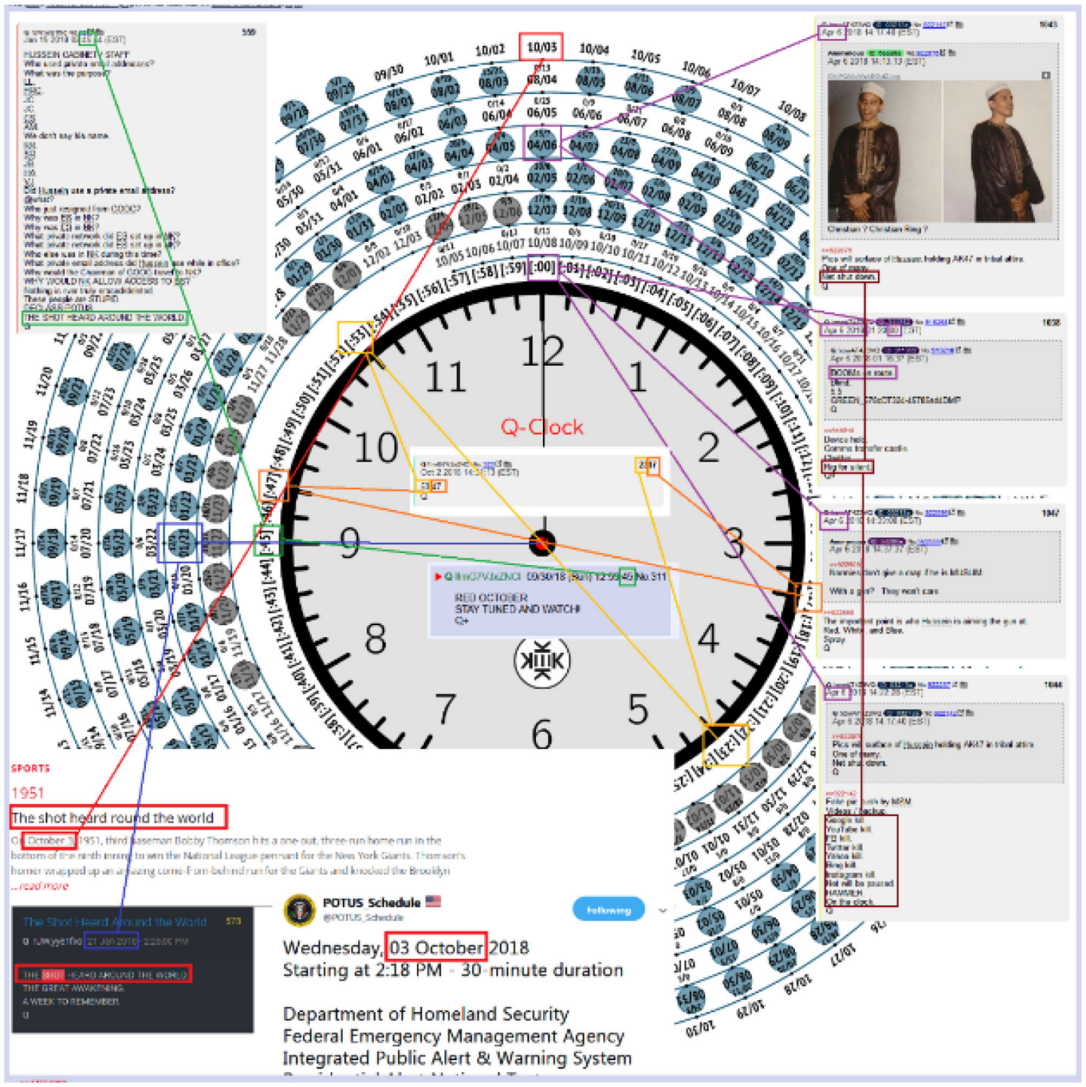
4chan Q-Clock.

Further, sometimes data is far too complex or difficult to reproduce. Take, for example, the screenshot (Figure 5) taken from a QAnon board on 4chan. The figure depicts a “Q-Clock,” a tool QAnons use to decode posts on mainstream social media platforms as well as “Q Drops” (messages from Q). As a researcher, how would I reproduce this Q-Clock while staying true to its original meaning? Each line drawn and text circled has a unique meaning to the anon, and hundreds of circles appear on the clock with numbers and dates of unknown importance. The cryptic nature of QAnon communications makes it nearly impossible to reproduce their text and imagery.

Data fabrication also inherently privileges the position of the researcher. Social scientists have been calling for attention to power dynamics between researchers and the researched for decades (for example, Merton, 1972; Garfinkel, 1984; Collins, 1986). To change text (or other forms of data) based on our own interpretations is to exert a form of power. It insinuates the researcher fully understands the original meaning and can alter it in a way that they see fit for the purposes of their research. We come to our research sites with various biases and unknowns, none of which can be avoided when fabricating data. This process of reproduction is dangerous not only because a researcher might misinterpret meaning, but also because it opens the door to unfair and biased representations.

Researchers might also unknowingly reproduce power dynamics should they fabricate content of a racist, sexist, or otherwise discriminatory nature. QAnons are known for hateful content, so to reproduce it as a researcher can become problematic. The reconstruction of anti-Black or antisemitic posts might reproduce harmful biases in the words of the researcher. It can be argued these kinds of hateful posts should be avoided entirely, so as to not amplify hateful messages to broader audiences. The ethics of this kind of direct reproduction should be an ongoing debate. Yet it seems a closed case that reconstructing an anti-Black post in the researcher's own words, for example, is an unethical practice.

A third and more logistical problem concerns the form of media researchers might want to fabricate. People do not simply communicate through text on social media. They use images, videos, audio, memes, GIFs, articles, hashtags, and more. How can a researcher fabricate a meme? An audio clip? While data fabrication might work for text, it does not hold up for various other mediums used frequently by social media users. Fabrication only seems to hold up for text-based data.

The lurking debate can be summarized as follows: Searchability and traceability are made easier online, so many researchers have decided to paraphrase text, videos, and audio while maintianing meaning. According to certain scholars, the practice of data fabrication may not sufficiently safeguard the anonymity of participants, thereby prompting a preference for qualitative data summaries or limiting direct quotations to public figures. The solutions I engage in my research are fairly straightforward. I ask for permission and provide participants with as much information as possible on the potential risks with special attention to how the nature of the internet changes the types of risks involved. I build transparent relationships with participants. The participant can then decide if they want their data to be used in a publication available to widespread audiences, and further if they want their name or username to be anonymous. Traceability and searchability should be discussed with participants candidly, and I have done this through direct messages and in Zoom interviews. This process has involved more steps with data that falls farther on the “private” side of the spectrum. For example, when a post is within a group with only a few members and I have to apply to join, I check in with participants at multiple points throughout the study. At first I ask if I can store their data, then again when I am considering using it in publication (at which point issues surrounding searchability can be discussed), and again when I know where the data will be published. I do not anonymize or request permission for posts that fall farther on the “public” side of the spectrum, such as a post on a mainstream platform by a celebrity. The case of QAnon reveals how difficult it can be to reproduce or fabricate complicated digital artificats of different mediums, and the power dynamics inherent in reproducing data of a racist or otherwise hateful nature. Further, QAnon has various subcultures which make it difficult for the researcher to have enough cultural knowledge to reproduce data.

Another solution is description rather than fabrication. Researchers might opt for a qualitative account of the posts they come across if they believe using the original post in a publication might harm the participant or themselves. With this method, researchers do what qualitative studies are known for: providing narratives. Although the narrative form is also subject to biases, misinterpretations, and power dynamics, it does not tell the reader: “This is the same as what my study participants produced.” Narratives might be most suitable for those studying vulnerable populations.

## 6. Institutional review boards

Institutional review boards (IRBs) affiliated with universities typically provide the first–and sometimes only–ethical analysis of research conducted on human subjects in the United States. Their work is integral to the research enterprise. First established in the 1970's, American IRBs were created to ensure the welfare and protection of human subjects involved in in-person research. It was only in the 1990's that IRBs began to oversee research in the social sciences and humanities, and the model for evaluation was still heavily based on biomedical research (Babb, 2020). IRBs are also notoriously misaligned with qualitative research because of excessive standardization (Babb et al., 2017). As a result, IRBs are not particularly well equipped to evaluate social science research conducted largely or exclusively online.

Kitchin (2007)'s guidelines on web-based research in Canada heavily influenced how Canadian IRBs classified submissions (Seko and Lewis, 2017). Kitchin determined that “non-intrusive” web-based research, by which they meant research using publicly accessible online material, did not constitute human subjects research and should therefore not be reviewed by IRBs. Text itself in cyberspace was not “human” enough. Some guidelines even indicate internet research is by definition public (see, for example, ESRC, 2015). Others think the IRB itself is non-sensical. Markham et al. (2018, p. 3) claims: “It is impossible to standardize or universalize what constitutes the ethically correct actions in technology design and research contexts.” Regardless of justification, many scholars view the involvement of IRB in internet-based research unfavorably.

On the other hand, some contemporary scholarship argues the IRB process is not enough. The digital landscape has changed the nature of research, and IRBs need to adapt (Miller, 2012; Bailey, 2015). For example, Bailey (2015) argues social media users are not the traditional infantilized subjects that IRBs assume researchers are dealing with in offline contexts. It is also particularly difficult to clarify the nature of an online field site (Boellstorff et al., 2012). Further, traditional conceptions of informed consent are grounded in offline research yet internet researchers have far less control over the lifetime of their data and therefore cannot be fully forthcoming with participants about potential risks (Matzner and Ochs, 2017). Despite the changes brought about by the internet, IRBs tend to lack the proper resources and processes for reviewing online research. Hutchinson et al. (2017) emphasize that most IRBs have little or no training in the ethical review of internet research. Seko and Lewis (2017) similarly claim American IRBs have no specific internet research ethics training nor guidelines for internet protocols. IRBs are also not equipped to understand the public vs. private nature of social media interactions (Hutchinson et al., 2017), an important conundrum discussed above.

A few internet researchers have suggested expanding the scope of IRB oversight. Luka et al. (2017) argue the IRB should be involved in more than just the proposal stage, as ethical considerations are needed at every stage. Hutchinson et al. (2017) suggest the IRB should be active throughout the entire research process including the dissemination of findings. This approach makes sense if we consider ethics as *choices* at critical junctures (Markham et al., 2018).

Important questions are still unanswered. Clearly not all internet interactions are public, but how should the researcher and the IRB determine what is public vs. private? If we take the approach that we should only use data when the participants consider their activity to be public, how do we know what participants are thinking when they post? Further, given IRBs remain embedded in different policy frameworks, national cultures, histories, and instititions (Babb, 2020), how do you account for differences when internet users can come from anywhere? How does a researcher identify themselves to a whole group if there are upwards of 100,000 members? Should our ethical standards change if the population we are studying is vulnerable?

I suggest some answers to these questions in the section following. Our decisions as researchers have implications for the endeavor of social science itself. Some practices such as deleting data can cause issues for replicability, reproducibility, and can introduce bias (Tromble and Stockmann, 2017). Sharing data is an important part of the process of ensuring valid research (Weller and Kinder-Kurlanda, 2017). So how do we ensure ethical practices while maintaining the integrity of the research process?

### 6.1. QAnon and institutional review boards

This section builds on my own experiences with the IRB for a project on QAnon activity on social media. Scholars have mixed opinions as to the role of the IRB. Should all social media research be exempt because it is technically public? Should the IRB be *more* involved and intervene at multiple stages of the research process? Should there be internet-specific protocols? First, I discuss the ways in which current review processes are ill-equipped to evaluate social media research. Next, I suggest what might be done to ameliorate the issues debated by scholars.

One of the first questions I was asked by the IRB was whether or not any participants will be located outside of the United States. This is done because privacy and consent laws vary considerably by country. However, national boundaries are not so simple on the internet. In most cases, the researcher has no way of ascertaining where participants are coming from. Perhaps more importantly, there is no way to know the age of participants. Minors and other vulnerable populations are likely to be included in most social media research. This is particularly true for research on the far-right where users are often anonymous. Even if a user claims to live in the United States and be of a certain age, there is no way to verify the accuracy of their statements. For example, I conducted a phone interview with a QAnon adherent named “Sean” who claimed to be 40 years-old and living in New Hampshire. I later Googled his username and discovered he was 64 years-old, living on the other side of the country, had an entirely different name, and was a convicted felon. Social media researchers can never claim to know where their participants are coming from or who they really are.

**Figure 6:**
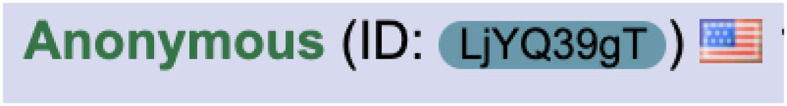
4chan user identification.

Consent procedures are problematic as a result. One of the most crucial tasks of IRB reviewers is to determine how consent will be obtained. Yet how can a researcher provide informed consent when they do not know their participants? What happens when researchers observe groups with hundreds of thousands of users? How can researchers avoid obtaining data from minors? Moreover, it can be incredibly difficult to contact users to ask for consent in the first place. My own experiences asking 4chan and 8kun users for consent to use their posts has been incredibly difficult. All users are anonymous by default on these platforms and there is no tool to directly message someone (like there is on Facebook and Twitter). See Figure 6 for how users are identified on 4chan. Everyone is labeled “anonymous,” assigned an ID number, and identified by their country's flag. Although I can be certain of where the user is posting from because the platform uses IP addresses to assign a flag, I have no sense of the user's name, age, race, ethnicity, or anything else of importance to researchers. Consent has become increasingly complex as people across the world take more of their lives online.

Another question asked by IRBs concerns the type of information obtained from participants. The IRB for my institution currently provides the following guidelines to determine private vs. public: “Private information is information that an individual can reasonably expect will not be made public. Generally, data sets that require specific permission from the data owner or are restricted access are considered ‘private.”' Just as there is no way to determine who an individual user is, there is also no way to determine what they expect will be made public and what they expect will be private. Next, what constitutes “specific permission” on social media? Is clicking “join group” asking for specific permission to view the data? There are various levels of restricted access which vary by platform. The private vs. public binary does not address the complexities of social media research nor does it tell IRB evaluators much about a proposed study.

The IRB also asks for a description of the overall methodology of the study including how the researcher will gain access to the communities they would like to study. Scholars have noted IRB evaluators are not formally trained on social media research protocols and specifically lack knowledge of the practices included in digital ethnography. Gaining access might mean creating an account. It could also mean directly messaging a group administrator and filling out a questionnaire to be accepted. Digital ethnography also changes the nature of participant observation and interviews in important ways. For example, I can scrape the data from hundreds of thousands of users on Twitter fairly easily and then publish whichever posts I deem necessary for my research. I am then confronted with the searchability and traceability of the posts.

The adequacy of IRBs in assessing social science research, particularly online research, has been a topic of debate among scholars. Some contend that web-based research universally entails publicly accessible data, negating the need for IRB intervention, while others suggest more involvement throughout the entire research process. The matter of how the IRB determines the distinction between public and private data remains unanswered.

There are no universal solutions to the problems IRBs face in regards to digital ethnography. How can we protect hundreds of thousands of unknowable users? Put simply: we cannot. Yet there are some practical steps researchers can take to ensure minimal risk to participants. First and foremost, social media researchers should almost never claim to know specific demographics of all of their participants. They need to make clear what they are able to find out and what they cannot know. I am particularly careful with descriptions of my participants in the discussion of my QAnon data because of the lack of certainty. Accordingly, it might be useful for social media researchers to engage in ethical practices as if all of their participants are vulnerable. This would mean taking extra care to de-identify data and avoiding particularly sensitive questions. Second, IRB evaluators should be aware of the nuances of ethnography adapted to online worlds in order to competently evaluate the potential risks of the research. Third, as the case of QAnon shows, understandings of privacy, consent, and identity are highly variable, and IRBs should be sensitive to these variations. It would be valuable and fruitful for IRBs to engage digital researchers to develop protocols more sensitive to online research.

## 7. Conclusion

This article has addressed three ongoing debates within the literature on digital ethnography: the public-private binary, lurking, and data fabrication. It has also discussed issues surrounding institutional review boards and their evaluation practices. Overall, I argue ethical digital ethnographic practice requires considering the nuances and complexities of our online social worlds. There are no universal rules which can be applied to all studies. There are, however, guidelines which can be followed and applied to diverse circumstances. My findings have implications for how digital ethnography should be conducted as our lives become even more enmeshed with digital worlds.

It makes little sense to designate all social media content as “public.” Instead, public is a determination which can be made by keeping in mind how difficult a space is to access. The broader the audience which can access the content, the farther it falls on the public side of the spectrum. The more barriers there are to access, the farther it falls on the opposite side of the spectrum. These barriers will look different across and within platforms. Researchers will need to make difficult decisions when deciding what data they should analyze and publish. It is important to keep in mind the various levels at which data exist, and to be in conversation with participants when questions arise. Treating privacy as a spectrum of accessibility and direct communication with participants about content publication are key to ethical practice.

Lurking has been a concern for decades. The first digital ethnographers had to grapple with the invisibility of their presence, and today it has become even more of a concern with the various tools available to hide one's identity. Although not everyone will choose to use their real name and information, it is imperative researchers make their presence known. This practice gives participants the right to decline, and also more generally raises awareness of who they choose to share their information with. Moreover, active participation benefits the researcher in that they are more deeply immersed in the space and better able to understand their participants.

Data fabrication is perhaps the most controversial practice. Many prominent ethnographers have chosen to reconstruct data in their publications. As I have shown, this practice leaves room for bias and misinterpretation, privileges the position of the researcher, and can even reproduce harmful ideas. Researchers should instead use data in its original form with direct, informed consent from the content producer. Content producers should be fully aware of searchability issues and other concerns unique to the affordances of the internet. Additionally, those who conduct research on extremist groups will likely encounter violent rhetoric and open threats directed toward public figures, margnizalized communities, opposing political parties, and other groups. Some threats may be direct and indicate imminent danger to a person or group. These should to be reported to proper authorities. Other threats come in the form of vague but violent rhetoric. The researcher then bears responsibility to expose the nature and degree of this form of violence to wider communities through various channels. While publishing or reconstructing this kind of rhetoric might reinforce harmful views, it may also serve to educate general publics on the reality of these spaces and act as a push toward regulation and other protective measures. Open discussion of these sensitive issues needs to take place to realize meaningful change.

Institutional review boards should require evaluators to do specific training on internet research, and questions need to be tailored to the complexities of digital space. In-person and online researchers are confronted with different ethical quandaries. Their proposals should reflect these differences. And although the internet might make it difficult to answer questions especially in regards to the identity of participants, researchers need to be forthcoming about what they know and do not know.

Many of the above suggestions can thought of through the lens of *relationships* and *local context*. Ethnographers have been grappling with their positionality vis-à-vis their subjects as well as the influence of social and cultural differences from the very beginning. In Geertz's (1973) famous discussion of “thick description,” he argues the entire enterprise of ethnography is predicated on analysis of our different webs of significance. In *Tally's Corner*, Liebow remarks that ethnography is defined by “local place stories” (Liebow, 1967, p. xiv). Duneier (1999) reflects on the power dynamics inherent to his relationships with his participants of different races, classes, and statuses. Yet he draws on his embeddedness within these relationships to portray an accurate and ethical account of his experiences. Questions of positionality and locality are particularly complex in digital space because of the ease of lurking and the global reach of the internet. This article draws on decades of reflection within traditional ethnography and argues we must continue to prioritize the ethnographic relationship and the importance of context in digital ethnographic research.

Many questions remain as to the ethics of digital ethnography. Are there types of posts or even groups which should be avoided by researchers entirely? What benefits and harms come from using real names and photos? Do some ethical practices affect the quality of the data, and if so what can be done to ensure data integrity? How can we reasonably make claims about groups or individuals in the absence of identifiable information? If researchers choose to take digital ethnography offline and get to know their participants in person, should a different set of ethical practices be applied? Researchers will need to contend with these and other questions as digital ethnography becomes more widely accepted.

## Data availability statement

The raw data supporting the conclusions of this article will be made available by the authors, without undue reservation.

## Ethics statement

The studies involving human participants were reviewed and approved by New York University Institutional Review Board, IRB-FY2022-6722. The patients/participants provided their written informed consent to participate in this study.

## Author contributions

MC contributed to the conception and design of the study, collected, organized and analyzed the data, wrote all drafts of the manuscript, revised, read, and approved the submitted version.

## Footnotes

^1^Operation Q has accounts on 38 different social media platforms and a following of hundreds of thousands.

^2^Interview data is not included in the findings, but I mention the process to give the reader a better sense of the methods within digital ethnography.
